# Multi-faceted quantitative proteomics analysis of histone H2B isoforms and their modifications

**DOI:** 10.1186/s13072-015-0006-8

**Published:** 2015-04-22

**Authors:** Rosalynn C Molden, Natarajan V Bhanu, Gary LeRoy, Anna M Arnaudo, Benjamin A Garcia

**Affiliations:** Department of Chemistry, Princeton University, Princeton, NJ 08544 USA; Department of Biochemistry and Biophysics, Epigenetics Program, Perelman School of Medicine, University of Pennsylvania, Room 9-124, 3400 Civic Center Blvd., Bldg. 421, Philadelphia, PA 19104 USA

## Abstract

**Background:**

Histone isoforms and their post-translational modifications (PTMs) play an important role in the control of many chromatin-related processes including transcription and DNA damage. Variants of histones H2A and H3 have been studied in depth and have been found to have distinct functions. Although 13 somatic histone H2B isoforms have been identified by various biochemical and mass spectrometric (MS) approaches, the distinct roles of these isoforms within human cells are as yet unknown. Here, we have developed quantitative MS techniques to characterize isoform-specific H2B expression across the cell cycle, in differentiated myogenic cells, and in different cancer cell lines to illuminate potential functional roles.

**Results:**

Using the MS strategies that we developed, we identified differences in H2B isoform levels between different cancer cell types, suggesting cancer or tissue-specific H2B isoform regulation. In particular, we found large variations in the levels of isoforms H2B1B and H2B1M across the panel of cell lines. We also found that, while individual H2B isoforms do not differ in their acetylation levels, trends in the acetylation on all H2B isoforms correlated with acetylation on other histone family members in the cancer cell line panel. We also used the MS strategies to study H2B protein expression across the cell cycle and determined that H2B isoforms that are alternatively spliced to carry a polyadenylation signal rather than the standard histone downstream element are expressed independently of the cell cycle. However, the level of protein produced from the polyadenylated transcripts does not contribute significantly to the total pool of H2B isoforms translated across the cell cycle or in non-cycling myogenic cells.

**Conclusions:**

Our results show that H2B isoforms are expressed at varying levels in different cells, suggesting isoform-specific, and possibly cell-type-specific, H2B gene regulation. The bottom-up mass spectrometry technique we developed for H2B quantification is compatible with the current standard histone H3 and H4 bottom-up ‘one-pot’ analysis platform so that H2B isoforms and their modifications can be studied in future experiments at the same time as histone H3 and H4 modifications. Therefore, we have expanded the histone landscape that can be interrogated in future experiments.

**Electronic supplementary material:**

The online version of this article (doi:10.1186/s13072-015-0006-8) contains supplementary material, which is available to authorized users.

## Background

Histones and 147 base pairs of DNA are together the building blocks of nucleosomes, which form the basic repeating units of chromatin. Chromatin is further folded and packaged into higher order structures with increasing degrees of compaction [1]. How tightly chromatin is packaged influences how accessible it is to DNA binding proteins, including transcription factors and DNA repair proteins [2]. Histones play important roles in regulating chromatin structure and function through dynamic post-translational modifications (PTMs) and specialized histones called histone variants.

There are five types of histone proteins: H2A, H2B, H3, H4, and H1, and with the exception of H4, all have multiple isoforms [3]. These isoforms are proteins encoded by separate genes that have the same core histone structure with some sequence variation [4]. Variants differ from canonical histones in their protein-protein interactions, localization in chromatin, number and types of PTMs, nucleosome stability, and tissue-specific expression profiles [5]. All of these differences lead to unique functions for histone variants compared to canonical histones. Histone variants are expressed in a DNA replication-independent fashion, are maintained at steady levels throughout the cell cycle, and are incorporated into nucleosomes outside of S-phase [3,6]. Some new histone variants have been discovered recently, but the most well-characterized core histone variants include H3.3, CENP-A, H2A.Z, H2A.X, and macroH2A [3]. Loss of function of any of these histone variants leads to acute consequences in specific cellular and developmental processes, such as fertility, proper centromere formation, genome integrity, and the DNA damage response [3]. Knockouts of variants H2A.Z and H3.3 are lethal in mice, and H2A.Z-knockdown embryonic stem cells have a decreased efficiency in self-renewal and differentiation [7-9].

**Figure 1:**
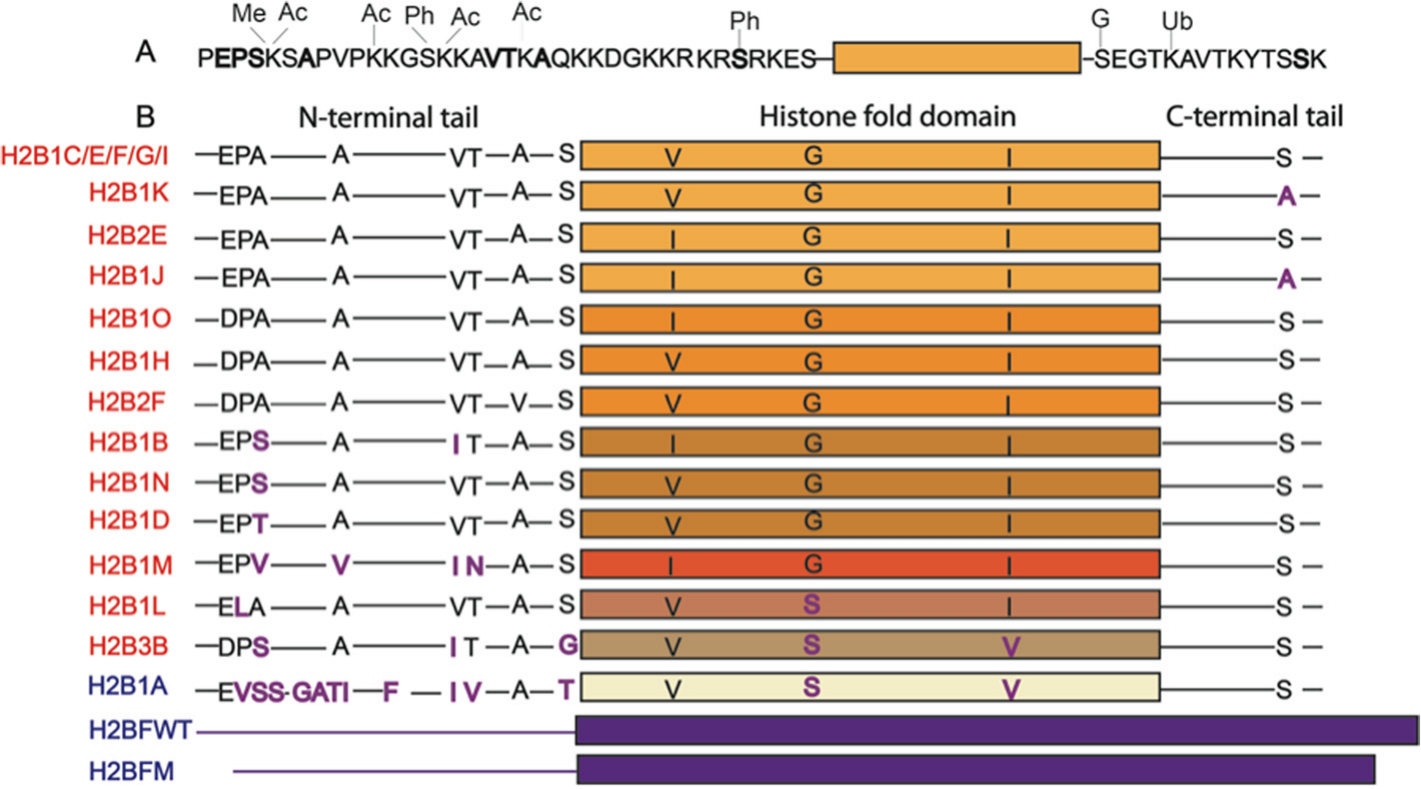
Points of sequence variation and modifications on human H2B isoforms. **(A)** Modification sites on the H2B N-terminal tail and C-terminal tail. Sites of sequence variation are in bold. **(B)** Sequence alignment of H2B isoforms. Points of sequence variation are highlighted in purple. Somatic isoforms are listed in red, and testis-specific variants are in blue. Variants H2BFM and H2BFWT differ significantly from other H2B isoforms across their entire sequences. Me = methylation, Ac = acetylation, Ph = phosphorylation, G = glycosylation, Ub = ubiquitination.

Most of the knowledge about histone variant functions comes from studies on H2A and H3, but very little is known about H2B isoforms. H2A and H3 isoforms have important functions in chromatin biology, and since there are so many H2B isoforms, it is likely that some of the H2B isoforms have unique functions. There are 16 isoforms of H2B in humans (Figure 1), 13 of which are expressed in somatic cells [10-12], and 3 of which are testis-specific [13,14]. The only human isoforms that have been studied in depth so far are the testis-specific variants H2BFWT and H2B1A [13,14].

Somatic human H2B isoforms are all encoded by genes located in histone cluster 1 on chromosome 6 (6p21-6p22), and in cluster 2 (1q21) and cluster 3 (1q42) on chromosome 1, and produce mRNA with characteristic 3′ stem-loop sequences and histone downstream elements [15]. H2B genes in the gene clusters are arranged in pairs with divergent H2A sequences that have a shared promoter region [16]. The histone genes inside of these clusters are all expressed at high levels during S-phase due to stabilization of transcripts with 3′ stem-loops [17]. Although expression of genes within the histone clusters is coordinated during the cell cycle, individual H2B (and H2A) genes are regulated by different promoter sequences and are expressed at different levels [15,16]. Somatic human H2B isoforms have very little sequence variation - they differ by as few as two amino acids and up to five amino acids (Figure 1). Most of the changes in sequence are small, but a few could affect protein function. For instance, the proline-to-leucine conversion in H2B1L would change the structure of the H2B N-terminal tail. In addition, there are many serine-and-threonine-to-alanine conversions in H2B isoform sequences, including at S36 in H2B3B, a site that is important in cell stress response regulation [18]. This serine-to-alanine difference occurs at the DNA-histone contact and could therefore potentially change nucleosome structure. In addition, differences in amino acid sequence could also be involved in isoform-specific protein-protein interactions. Small differences in known histone variants are responsible for variant-specific chaperone and chromatin remodeling enzyme recognition [19,20].

There is evidence for somatic H2B sequences having unique functions in other organisms. H2B variants in *Trypanasoma brucei* and in *Plasmodium falciparum* form dimers specifically with the H2A variant H2AZ and localize to active genes [21,22]. Most recently, it was discovered that the H2B variant H2BE is involved in controlling olfactory gene expression in mice, suggesting that some somatic H2B variants may have tissue-specific roles [23]. This H2B variant has an analogous sequence in humans (H2B1L). In fact, H2B sequence variation is conserved among higher eukaryotes [24], and some of the variants characterized in other organisms may have counterparts with similar functions in human cells.

The sequence similarity between histone H2B isoforms makes it challenging to differentiate between isoforms at the protein level. Mass spectrometry (MS) methods for identifying histone proteins include top-down MS of intact proteins, middle-down MS of large peptides, and bottom-up MS of smaller peptides. Two previous investigations, by Siuti *et al*. and Bonenfant *et al*., used top-down and bottom-up MS to identify H2B isoforms in human cell lines [10,11]. Siuti *et al*. used top-down to identify 8/13 H2B isoforms; they also used l-methionine-methyl-^13^C-methyl-*d*_3_ to label potential methylation sites and histone deacetylase inhibitors to increase levels of H2B acetylation. They found no evidence of H2B methylation but did identify two acetylation sites on the most abundant H2B isoform [11]. Bonenfant *et al*. used a variety of protease digest methods, including Glu-C digests, to identify 11 histone H2B isoforms and 3 acetylation sites [10]. These studies confirmed that many H2B isoforms are present at the protein level for the first time but failed to detect some lower abundance H2B isoforms.

To date, methods to comprehensively, consistently, and quantitatively analyze histone H2B isoforms and their post-translational modifications have not been thoroughly compared and established. Here, we describe top-down and bottom-up mass spectrometry methods we developed to identify all 13 known H2B isoforms. The bottom-up method, unlike previous strategies, uses a well-established chemical derivatization method to create H2B N-terminal peptides of a similar and consistent length that can be resolved reproducibly using reversed-phase chromatography. As a result, this method can be used to quantify the 9 out of 13 somatic H2B isoforms that have unique N-terminal sequences and their modifications.

Since there is little research on potential biological differences between somatic H2B isoforms, we used the mass spectrometry methods developed to profile H2B isoform levels from mammalian cells. These top-down and bottom-up MS methods were utilized to profile the H2B isoform composition of different cancer cells. If H2B isoforms are regulated differently across cell types, then it is possible that some isoforms have unique cell-type-specific functions. We also used these methods to determine whether some H2B isoforms are expressed independently of DNA replication and to determine whether there are differences in the rates of synthesis of H2B variants between proliferating and terminally differentiated cells.

## Results

### Top-down MS analysis of H2B

**Figure 2:**
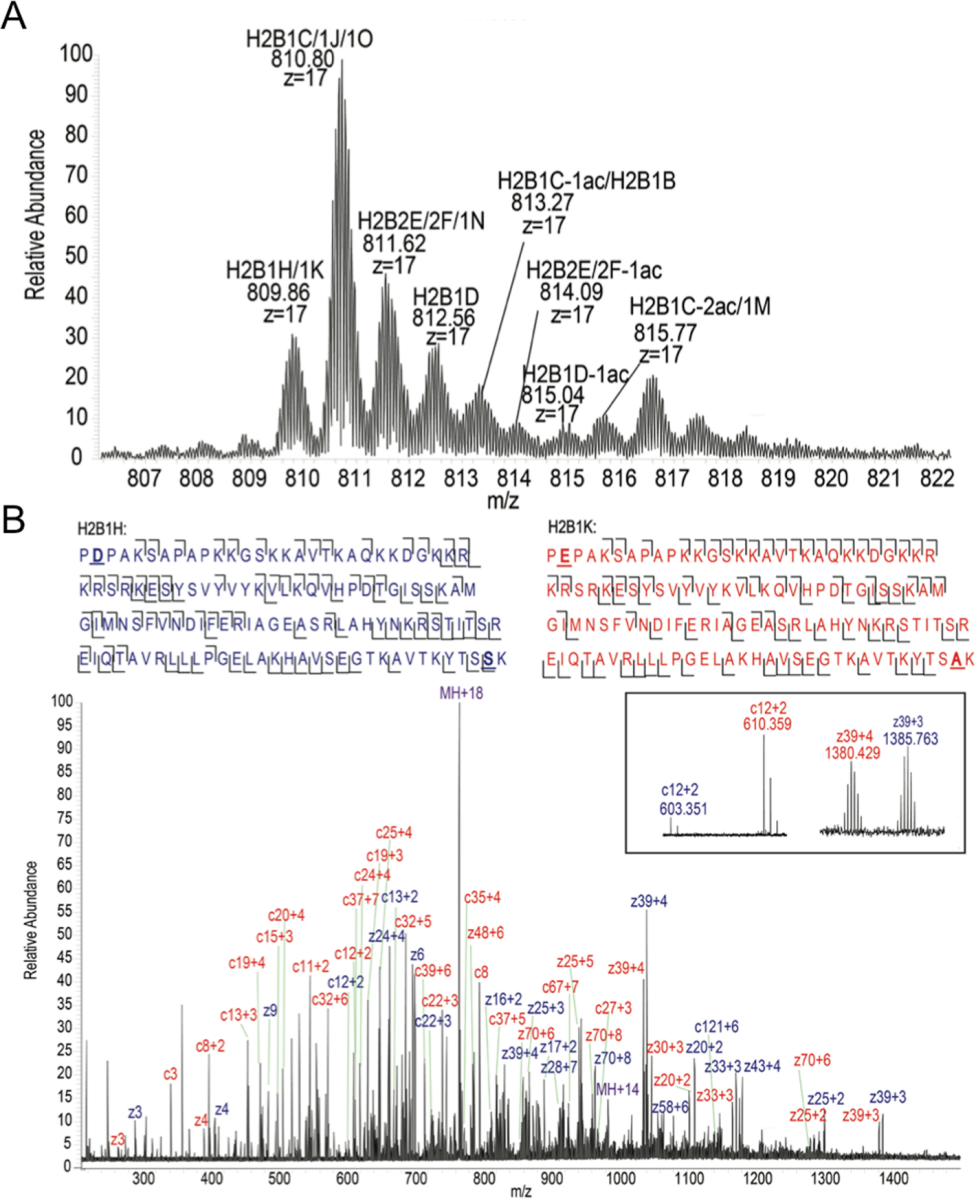
Top-down MS of histone H2B isoforms. **(A)** Intact mass spectrum of HPLC-purified Jurkat H2B at the most abundant charge state, *z* = 17. H2B isoforms were identified based on MS/MS spectra and intact mass. Ac = acetylation. **(B)** Fragmentation spectrum of H2B isoforms H2B1K and H2B1H. Identified c and z ions are marked in blue (H2B1H) and red (H2B1K), respectively. The sequences at the top of the figure illustrate the extent of sequence coverage based on identified c and z ions. The inset shows magnified spectra of select c and z ion peak pairs for H2B1H and H2B1K, respectively.

Top-down mass spectrometry of intact proteins is essentially the only method that can distinguish between all of the histone H2B isoforms and their PTMs, as it is the only approach that retains complete sequence information. We purified histone H2B from acid-extracted histones using C18-based reversed-phase chromatography (Figure 2A), then infused the purified H2B directly into the mass spectrometer and collected spectra to determine the intact masses of the H2B isoforms. Peaks from the H2B intact mass spectrum were then fragmented using electron transfer dissociation (ETD) for sequence analysis. We used ProSightPTM2.0 [25] and the MASH suite [26] programs as well as manual annotation to de-convolute masses from the H2B MS1 intact mass spectra and to identify H2B isoforms and sites of post-translational modifications from the ETD MS/MS spectra.

There are six main peaks in the intact mass spectrum of histone H2B (Figure 2A). The 5 most abundant masses match the predicted masses for 11 out of 13 known H2B isoforms (Figure 2A), as well as the monoacetylated forms of 3 H2B isoforms (H2B2F, H2B1D, and H2B1C) and the di-acetylated form of 2 isoforms (H2B1C and H2B1D). The low-mass peaks next to the main H2B peaks are artifacts caused by signal processing in the mass spectrometer. All of the H2B isoforms expressed in human somatic cells are the same length and have very few points of sequence variation. Consequently, many of them have the same intact mass or masses that are similar enough that they cannot be resolved based on the full-MS spectra alone (Additional file 1: Table S1). We identified these species at the MS/MS level using distinguishing *c* and *z* ions in their ETD fragmentation spectra. For example, histone H2B1K and histone H2B1H are proteins with masses that differ by only 2 Da and as a result have almost completely overlapping isotopic peak clusters. They differ in sequence by one amino acid at position 2 and at position 125 (Figure 1A). This sequence difference leads to unique *c* and *z* ion series for the two H2B isoforms (Figure 2B) in their combined fragmentation spectra. We did not detect two H2B isoforms (H2B3B, H2B1L) using top-down MS, despite prior evidence for these forms from real-time (RT)-PCR experiments [12]. These isoforms have similar masses to the most abundant H2B isoforms, so it is possible that their signal was masked by the more abundant species. We estimated abundances of the isoforms and modified forms based on the protein ion relative ratios (PIRRs) using the five most abundant isotopic peaks for each isotopic cluster (Additional file 1: Table S1) as described previously [27].

### Bottom-up MS analysis of H2B

Top-down MS is a powerful technique that can distinguish between the majority of H2B isoforms and their modifications; however, it is less sensitive and arguably less quantitative than other MS strategies. We developed bottom-up MS techniques to quantify and to identify H2B isoforms that were not abundant enough to be detected by top-down MS. As with other histone proteins, histone H2B is very lysine and arginine rich, making it difficult to digest with standard proteases and also making its digested fragments very hydrophilic and therefore not as compatible with standard reversed-phase C18-based chromatography as frequently utilized in proteomics experiments.

We analyzed H2B by derivatizing it first with propionic anhydride to cap primary amines on lysine residues with propionyl groups. This is a standard chemical derivatization technique that our lab uses to process histones [28], but we had not applied it yet to study H2B isoforms and their modifications. Capping the lysines prevents their digestion with trypsin so that the sequence is only digested after arginine in an Arg-C-like manner but with much more efficiency and specificity than using Arg-C itself. Propionylation also increases the hydrophobicity of histone peptides and thereby improves their retention and resolution on reversed-phase columns. Digesting after arginine creates a 29-amino-acid-long H2B N-terminal peptide encompassing the entire H2B N-terminal tail, the part of the H2B sequence with the most sequence diversity and modifications. It also creates a peptide encompassing the H2B C-terminal tail - the site of an important ubiquitination PTM [29].

**Figure 3:**
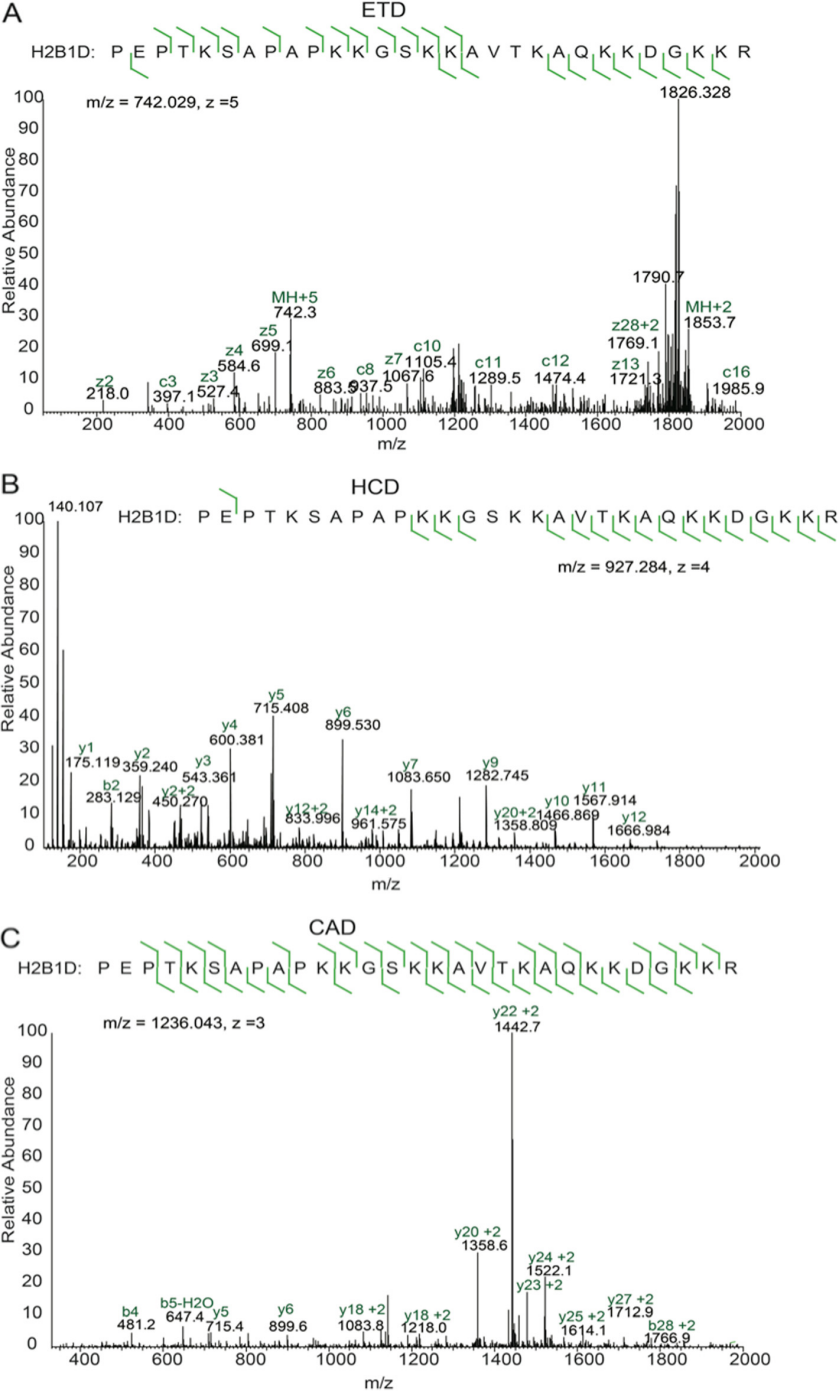
Comparison of MS/MS fragmentation methods for the propionylated H2B N-terminal tail. **(A)** ETD fragmentation spectrum of the H2B1D(1–29) sequence. **(B)** HCD fragmentation of the H2B1D(1–29) sequence. **(C)** CAD fragmentation spectrum of the H2B1D(1–29) sequence. CAD = collisionally activated dissociation, ETD = electron transfer dissociation, HCD = higher energy collisional dissociation.

The H2B(1–29) peptide created using this method is longer than the histone peptides that we usually analyze, and it contains 11 propionylated lysine residues, making it more hydrophobic. We were concerned about finding an appropriate fragmentation technique that would give us a complete ion series covering the entire tail sequence so that we could confirm sites of modification and sequence variation. We analyzed propionylated H2B peptides using three different fragmentation methods: collisionally activated dissociation (CAD), higher energy collisional dissociation (HCD), and ETD (Figure 3). All of these fragmentation methods created complete, or nearly complete, ion series across the H2B(1–29) peptide; however, each fragmentation method had a different optimal charge state. There were three abundant charge states of the H2B(1–29) peptide: the most abundant species is +4, followed by +3, and then +5.

CAD spectra were optimal for analyzing the N-terminal peptide sequence (Figure 3C). Fragmentation of both the +4 and +3 charge-state species created complete or nearly complete *y*-ion and *b*-ion series. There was a particularly strong *y* + 2 ion series for this peptide, with the most abundant fragment ions occurring on the N-terminal side of prolines. This proline-directed fragmentation was advantageous because it captures the sequence diversity of the H2B tail. This set of ions can be used to easily identify and distinguish between H2B isoforms. For example, the monoacetylated H2B1D N-terminal and the H2B1N N-terminal tail have different sequences but the same mass. The peaks can be distinguished from each other based on the characteristic proline-directed fragments in their MS/MS spectra (Additional file 2: Figure S1A and B).

After analyzing propionylated H2B samples, we identified H2B(1–29) N-terminal tail sequences representing seven unique H2B isoforms and two tail sequences that were shared between the remaining six H2B isoforms. In addition, each N-terminal tail sequence had up to a total of four lysines modified with acetyl groups (K5, K12, K15, K20). The most commonly acetylated residues, as determined by their unique fragment ion intensities, were lysine 12 and 15, which is in agreement with previous research which quantified H2B acetylation by Western blot [30]. We also identified a potential new N-terminal H2B ubiquitination site at lysine 5 (Additional file 2: Figure S1), in addition to the more commonly studied C-terminal H2B(K123) ubiquitination site [29].

**Figure 4:**
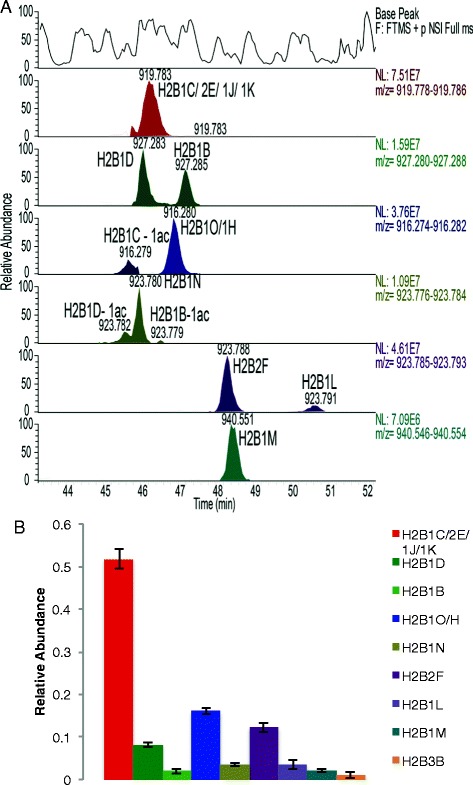
Propionylated H2B N-terminal tail chromatography and quantification. **(A)** H2B isoform N-terminal tails can be chromatographically resolved after propionylation. The top panel is the base peak chromatogram, and each of the following panels is an extracted ion chromatogram for different N-terminal peptide masses. Isobaric species such as H2B1D and H2B1B were completely resolved. **(B)** Relative quantification of H2B isoforms in HeLa samples. Mean ± SE, *n* = 5.

All of the unique H2B N-terminal tails, except for the nearly isobaric isoforms H2B3B and H2B1C, and their acetylated forms were chromatographically resolved on a C18-column after propionylation (Figure 4A). In addition, the H2B N-terminal tails elute later than most other histone peptides due to their size, making it possible to analyze H2B at the same time as histones H3 and H4 from an unfractionated histone preparation. Such separation enables quantification of H2B isoforms by peak integration of the extracted ion chromatograms for each N-terminal peptide mass. We divided the sum of the peak areas for each form by the total detected signal for all N-terminal tail peptides to give the relative abundances of each H2B isoform. The relative abundances for different H2B isoforms extracted from HeLa samples are given in Figure 4B. The relative abundance of H2B3B was quantified separately using a different peptide (EVQTAVR) that is unique to H2B3B. This method of quantification is very consistent, as demonstrated by the small standard error in relative abundance for each isoform, derived from five different HeLa samples that were run on different days and on different instruments and which were from separate histone preparations (Figure 4B). The relative abundance of acetylation on each H2B isoform can be obtained in a similar manner. These results show that the levels of acetylation do not differ significantly across H2B isoforms (Additional file 3: Figure S2).

### Profiling H2B isoform composition in commonly used cancer cell lines

Previous studies have demonstrated that there is variation in the expression of the mRNA of different H2B isoforms across cancer cell lines [12,31]. This suggests that there may be different abundances of H2B isoform proteins between cell lines and possibly cell-type-specific functions for the different H2B isoforms. Also, some H2B transcripts are frequently overexpressed in certain cancer types, so we would therefore expect to see some variations in their protein abundances as well [32,33]. We used top-down MS and bottom-up MS to confirm differences in H2B isoform protein levels across different cell lines, including a catalog of cancer cell lines that represent many different cell types and cancer types.

Top-down MS was used to confirm that there are differences in the levels of H2B isoforms across cancer cell lines (Additional file 4: Figure S3). Relative to the most abundant H2B1C/1 J/1O peak, we found the most variation in the H2B1H/H2B1K, H2B2E/2 F, and H2B1M levels. However, the top-down MS results were not as quantitative as the bottom-up results, and we were unable to deconvolute isobaric species contributions to the differences in abundances that we observed. So once we confirmed differences in H2B levels at the protein level using top-down MS, we also profiled H2B content in cancer cell lines using the bottom-up MS technique described in the previous section.

**Figure 5:**
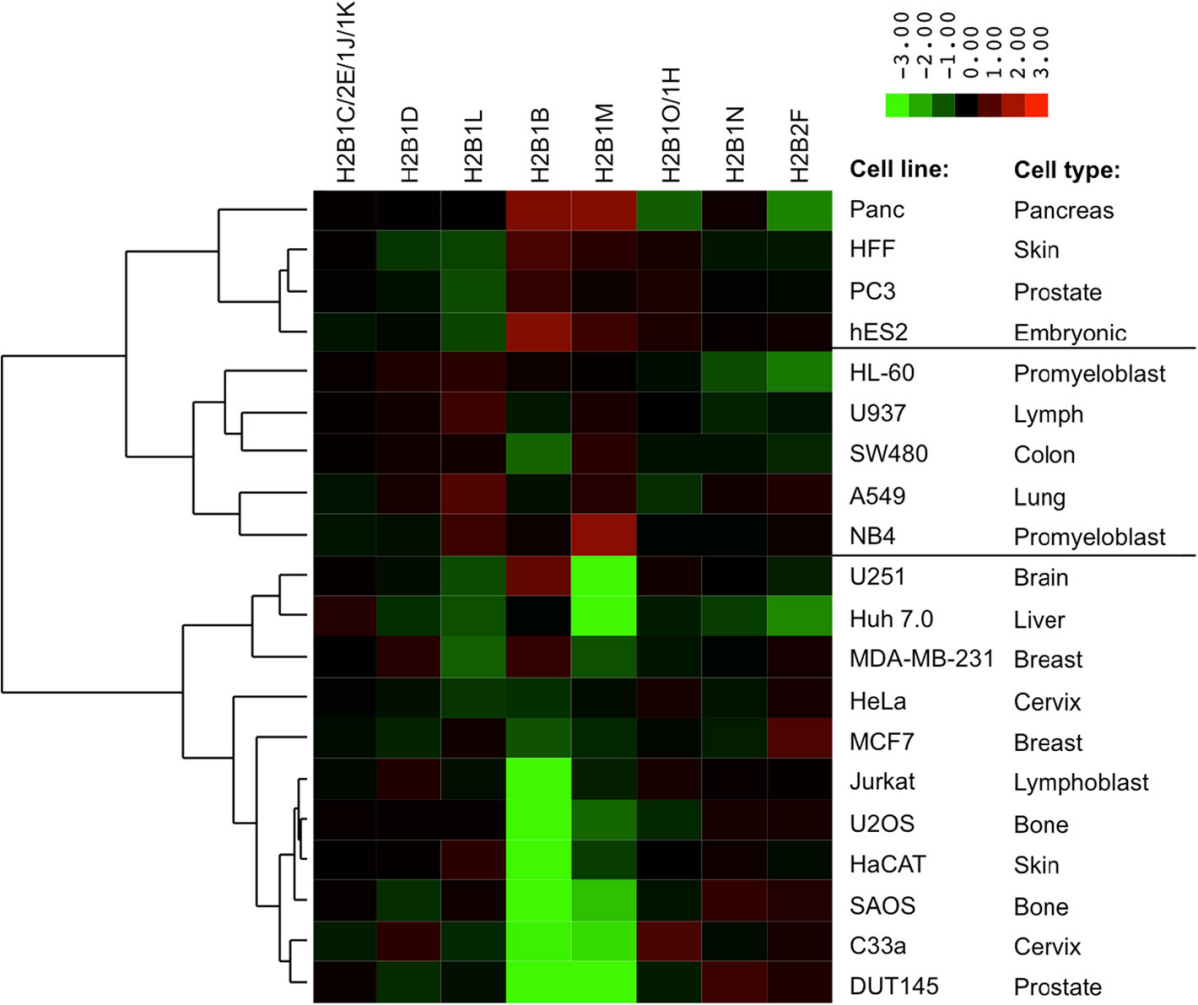
Hierarchical clustering of H2B isoform abundances across 20 cell lines. The relative abundances of H2B isoforms were determined using bottom-up MS, normalized against the average abundance for each isoform across all cell lines and log2 transformed. Data for H2B3B was excluded from the analysis because the characteristic H2B3B peptide (EVQTAVR) was missing from many MS runs due to poor chromatography at the beginning of the LC-MS gradient. The remaining H2B isoforms were quantified for each cell line based on the H2B(1–29) tail sequence and had CV < 30%, *n* = 3 or 4.

We determined the relative abundances of H2B isoforms (Figure 5), and acetylation levels (data not shown), across 20 cell lines representing different cell types using bottom-up MS. This panel of cell lines had previously been used to profile histone H3 and H4 PTMs [34]. The panel consisted of mostly cancer cell lines but also included human foreskin fibroblast (HFF) and human embryonic HES-2 cells. HFF and HES-2 are non-cancerous cell lines and have normal chromosome numbers and so can be used as reference samples where H2B isoform levels would not be influenced by chromosomal abnormalities. To compare H2B isoform abundances, we normalized the relative abundance for each isoform to the average abundance for that isoform across all of the cell lines. We then clustered cell lines with similar H2B isoform profiles using hierarchical clustering with Euclidian distance as a metric.

Similar to the top-down cancer cell line data, we observed differences in H2B isoform levels across the different cell lines (Figure 5). Cell lines from the same cell type clustered together. The only exceptions were the prostate cell lines PC3 and DU145 and the skin cell lines HaCAT (keratinocyte) and HFF (fibroblast), which had very different H2B isoform profiles. The most striking difference between cancer cell lines was in the levels of isoforms H2B1B and H2B1M. HFF and hES2 cells had higher levels of both isoforms compared to most of the cancer cell lines. In addition, one third of the cell lines profiled had significantly lower levels of H2B1B than the rest of the cell lines. Other H2B isoforms that varied in abundance between cell types, but did not show as striking differences as H2B1B and H2B1M, are H2B2F and H2B1L.

Most cell lines had very similar levels of H2B acetylation (approximately 11% of the total signal on average). The exceptions were hES2, Jurkat, and C33a cells, which had higher levels of acetylation (23%, 18%, and 22%, respectively, of the total signal), and SW480 cells, which had lower (6%) levels of acetylation. This result is consistent with a study from our lab that used the same cancer cell line data to analyze histone H3 and H4 modifications that also found high levels of histone acetylation in hESC and C33a cells [34]. The low levels of H2B acetylation in SW480 cells are in agreement with a previous study, which found that overexpression of RGC-32 in SW480 suppressed H2BK5 acetylation [35].

### Polyadenylated H2B mRNA and protein levels in the cell cycle and in terminally differentiated cells

Histone levels are very tightly regulated; in fact, excess histone synthesis is detrimental and leads to chromosome abnormalities and cell death [36,37]. Histone variants, such as macroH2A, H2A.Z, and H3.3, that are made outside of DNA replication have specific functions [3,7]. Polyadenylated H2B transcripts accumulate following p53-induced senescence and during terminal cell differentiation [12]. Therefore, we wanted to determine whether the polyadenylated H2B mRNA is expressed in a cell-cycle-independent manner and whether this leads to an increase in the levels of these proteins outside of S-phase. If these H2B isoforms are produced outside of S-phase, then it is possible that they have unique functions as replacements for canonical H2B. In addition, it is possible that H2B isoforms produced outside of S-phase associate with other replication-independent variants when they are assembled into chromatin. We hypothesized that polyadenylated H2B sequences would be expressed in a cell-cycle-independent manner. In order to test this hypothesis, we designed primers against the histone H2B1D coding sequence and against the polyadenylation sequence. We synchronized cells and synthesized cDNA from mRNA collected during G1- and S-phases then compared the relative amount of mRNA across these cell cycle phases using quantitative real-time (q-RT)-PCR.

**Figure 6:**
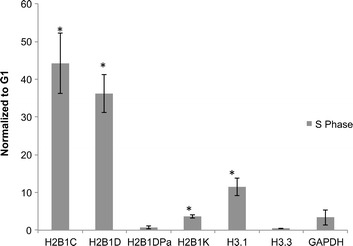
Polyadenylated histone isoforms show no changes in levels across the cell cycle. H2B mRNA relative expression in S-phase of the cell cycle compared to G1-phase. Canonical histones (H2B1C, H2B1D, H3.1) have much higher relative expression during S-phase compared to G1 than replication-independent histones (H3.3) and polyadenylated H2B1D. H2B1D results are from primers against the H2B1D coding sequence, and H2B1DPa results are from primers against the polyadenylation sequence. mRNA was synthesized using random hexamers and analyzed by q-RT-PCR. Bars marked with a ‘*’ indicate a *P* value <0.01, indicating that that histone variant was expressed at significantly higher levels in S-phase than G1-phase. Mean ± SD, *n* = 3.

The results of this experiment are shown in Figure 6. Cell cycle synchronization was validated by flow cytometry analysis (Additional file 5: Figure S4). The q-RT-PCR data was normalized against 18S rRNA. We analyzed the expression of polyadenylated H2B1D (H2B1DpA) and all H2B1D transcripts (H2B1D), as well as the expression of a known replication-dependent histone variant (H3.1), a known replication-independent histone variant (H3.3), and a histone H2B isoform with no polyadenylation signal (H2B1C) as controls (Figure 6). The results show that the replication-dependent histones H2B1C, H3.1, and H2B1D are expressed at much higher levels in S-phase compared to G1-phase, as expected. The replication-independent variant H3.3, on the other hand, shows little variation between the different phases of the cell cycle. As we hypothesized, the polyadenylated form of histone H2B1D showed a very similar trend to H3.3 expression throughout the cell cycle with very little variation between G1- and S-phases. Histone H2B1K is another H2B isoform that can be polyadenylated. We did not design primers against the polyadenylated form of H2B1K, but it showed less cell cycle variation with primers that recognize all H2B1K forms than other histone mRNA.

**Figure 7:**
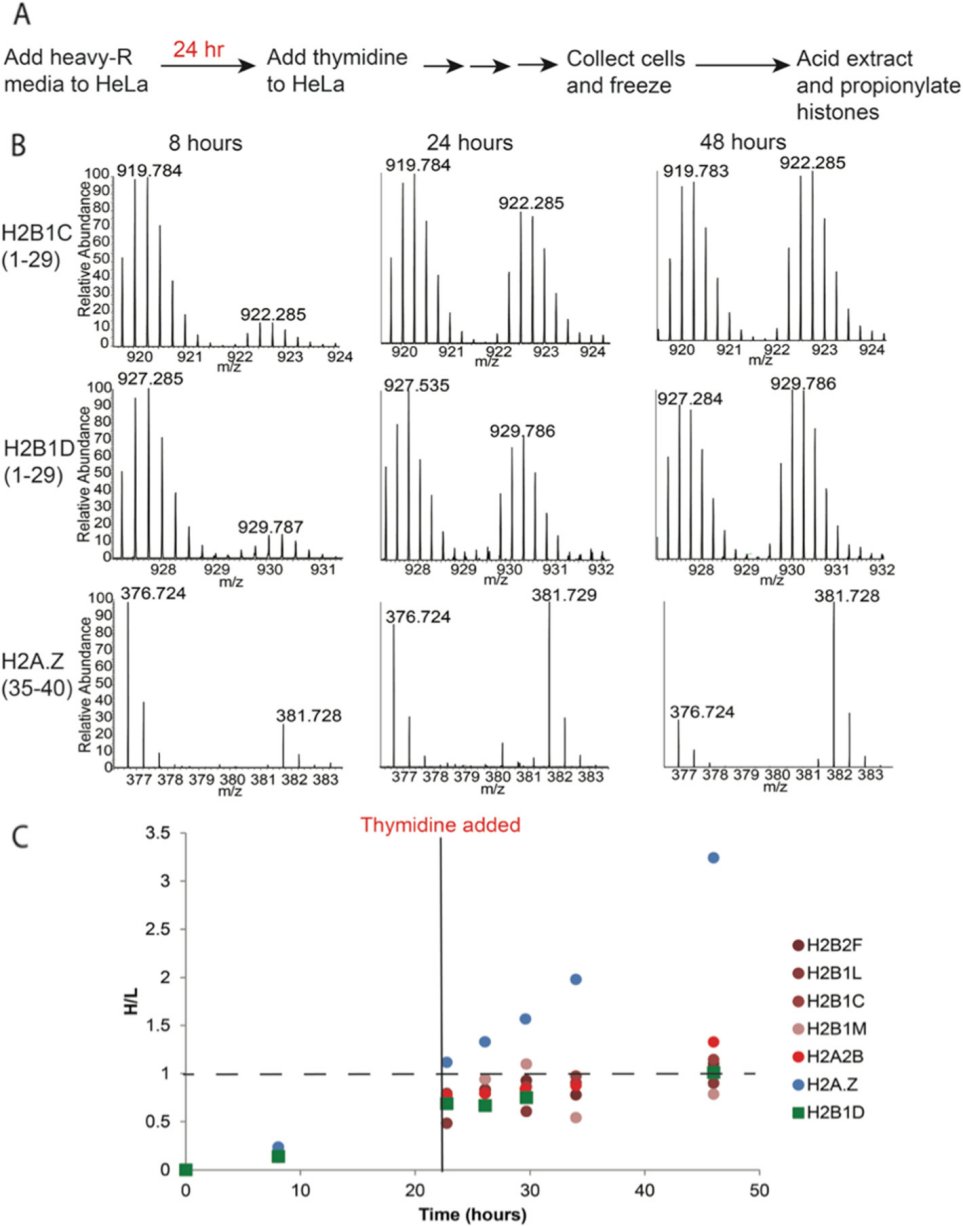
Heavy arginine incorporation into newly synthesized histones before and after blocking HeLa cells in S-phase using thymidine. **(A)** Cells were grown in heavy media for 24 h or one doubling so that the ratio of new-to-old histone (heavy-to-light) should be 1:1. They were then blocked with thymidine. Time points were collected, and histones were analyzed using bottom-up MS. **(B)** MS1 spectra of H2B1C/2E/1 J/1 K(1–29), H2B1D(1–29), and H2A.Z(35–40) showing increase in heavy-labeled ‘new’ protein over time. H2B1D(1–29) and H2B1C(1–29) synthesis is blocked, and so, the proteins stopped incorporating heavy arginine after the double-thymidine block at 24 h. The synthesis of replication-independent H2A.Z continued after the double-thymidine block. **(C)** Plot of heavy arginine incorporation over time for replication-dependent and replication-independent histone isoforms. H2A.Z (blue) is a known replication-independent isoform, and H2A2B is a replication-dependent isoform (red). The replication-independent H2A.Z isoform continues to incorporate heavy arginine following the thymidine block, but replication-dependent histones stop incorporating the label.

We wanted to confirm that the cell-cycle-independent expression of polyadenylated H2B mRNA also leads to cell-cycle-independent production of H2B proteins. If there is a significant expression of mRNA of specific H2B isoform outside of S-phase, then these isoforms should be made throughout the cell cycle. To test this, we pulse-labeled cells with heavy arginine (^13^C_6_^15^N_4_-Arginine) to track histone synthesis before and after blocking cells in S-phase (Figure 7A). The heavy arginine is incorporated into newly synthesized proteins, resulting in a 10-Da mass shift between new and old histone peptides (Figure 7B). First, HeLa cells were grown for one doubling time (24 h) in heavy arginine media so that half of the histones were labeled. At this point, we added excess thymidine to the growth media, which prevents DNA synthesis and blocks cells in S-phase. This block should also prevent replication-dependent histone synthesis, but not replication-independent synthesis. Therefore, after the thymidine block, replication-independent histones should continue to incorporate heavy arginine, but replication-dependent histones will not.

As expected, the known replication-independent histone variant H2A.Z continued to incorporate heavy arginine at the same rate after the thymidine block (Figure 7B) as before. Other replication-independent variants such as histone H1.x showed similar trends (data not shown). Heavy arginine incorporation for replication-dependent H2A (H2A2B) and H2B (H2B1C, H2B1L, H2B1M) histone isoforms with no known polyadenylation signal leveled off after the thymidine block close to the expected 1:1 heavy-to-light ratio. There was still some incorporation of heavy arginine because a portion of the cells was not completely synchronized after one thymidine block. The H2B isoform with a known polyadenylation signal, H2B1D, showed a trend that was indistinguishable from other replication-dependent histone isoforms. This indicates that the polyadenylated H2B1D mRNA did not contribute significantly to the H2B1D protein levels after the thymidine block.

**Figure 8:**
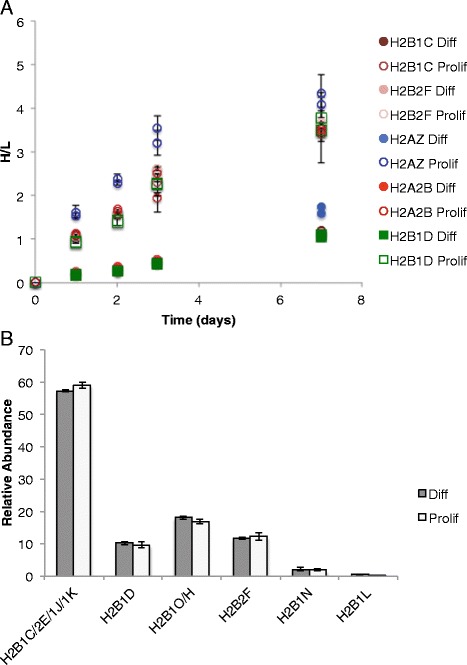
Histone H2B variant levels in myoblast cells compared to myotube cells. **(A)** Proliferating myoblast (prolif) and differentiated myotube cells (diff) were cultured in media containing heavy arginine, and samples were collected over the course of 7 days to monitor new histone synthesis. Heavy (new) over light (old) levels for histone variants in proliferating or differentiated cells were plotted over time for two biological replicates. **(B)** Relative abundances of histone H2B variants in myotubes (diff) versus (myoblasts). The abundance of each variant was summed and divided by the abundance of all of the variants. Mean ± SE, *n* = 3.

Studies have shown that polyadenylated replication-dependent variants such as histone H2B1D and H2A1C are present at very low levels in normal cycling cells but increase in terminally differentiated cells or senescent cells [12,38,39]. It has been suggested that polyadenylated histone variants are required in these non-replicating cells to maintain chromatin integrity [7,39]. In order to test whether elevated polyadenylated histones levels in terminally differentiated cells are incorporated into chromatin, we labeled human myoblast and myotube cell cultures with (^13^C_6_^15^N_4_-Arginine) over the course of 7 days (Figure 8, Additional file 6: Figure S5). We also analyzed the expression of different histone variant mRNAs, including polyadenylated H2B1D mRNA, in myotubes versus myoblasts by quantitative RT-PCR. Myotubes are non-cycling cells that are known to have higher levels of replication-independent variants than their proliferating progenitor cells, myoblasts [40].

Most histone mRNAs were produced at higher levels in the proliferating myoblasts compared to myotubes (Additional file 7: Figure S6A). This result was expected because the myoblasts are replicating, while myotubes have exited the cell cycle. Polyadenylated H2B1D mRNA, however, was produced at significantly (*P* < 0.05) higher levels in myotubes compared to myoblasts. This result is in line with other studies that found that polyadenylated H2B variants were produced at higher levels in non-cycling cells [12]. Since polyadenylated H2B1D is synthesized at higher levels in myotubes, we would then expect that more heavy, newly synthesized H2B1D would be incorporated into the chromatin compared to non-polyadenylated variants. We would also expect that there would be a higher percentage of H2B1D in the overall H2B profile of myotubes compared to myoblasts.

The overall rate of heavy arginine incorporation was much lower in non-dividing myotubes than in the proliferating myoblasts (Figure 8A). The histone variants H2A.Z and H1.x were labeled at a faster rate than all of the other histones under both proliferating and differentiation conditions, indicating a higher turnover (Figure 8A and Additional file 8: Figure S7). All of the canonical histone variants, including histone H2B, incorporated heavy arginine at essentially the same rate (Figure 8A). In addition, the relative abundances of different histone H2B variants remained essentially unchanged between proliferating and differentiated cells (Figure 8B).

## Discussion

Through a combination of different mass spectrometry methods, we identified all 13 H2B isoforms. We also identified H2B sequences modified with up to four acetylations, the known C-terminal ubiquitination site, and one new ubiquitination site. We described a bottom-up method that can be used to consistently quantify the relative abundances of nine H2B isoform N-terminal tail sequences and their modifications from unfractionated histone samples. This same method can also be used to study the H2B C-terminal tail and its modifications in a very straightforward and rapid manner.

There are variations in the levels of H2B isoforms and their modifications in different cancer cell lines. Variations in H2B isoform expression had been observed before in quantitative PCR (qPCR) assays and microarray studies at the mRNA level; however, this is the first investigation of variations at the protein level. H2B isoforms are all located in the histone gene clusters, facilitating their expression during S-phase, but individual H2B genes have different promoter sequences [41]. This means that individual H2B genes may be regulated differently in cancer cell lines and possibly in a tissue-specific manner. We found that cancer cell lines from the same tissue exhibited similar H2B isoform profiles, so it is possible that H2B isoforms exhibit tissue-specific profiles. The differences in H2B cancer cell lines may alternatively result from chromosomal aberrations or just from other abnormal gene regulation in cancer cells. PANC cells consistently have an extra chromosome 6 (containing histone clusters 1 and 2), U2OS has rearrangements of chromosome 1 (containing histone cluster 3), and HeLa cells have extra copies of chromosomes 1 and 6 (ATCC.org). Regardless, differences in H2B isoform expression between cancer cell lines should be investigated further as controlling levels of H2B may be important for maintaining histone levels and maintaining a balance between histone family members.

The most striking difference between the cell lines was in the levels of H2B1B and H2B1M. Histone H2B1B only differs from other H2B isoforms at two positions with an alanine-to-serine difference and a valine-to-isoleucine difference, and it is unclear what affect these sequence variations would have on protein function. However, H2B1M has much larger differences in sequence across four amino acids in its N-terminal tail, including a threonine-to-asparagine difference that is not present in any other H2B isoforms. Differential expression of H2B1B had not been identified before, but low-H2B1M expression was identified as a biomarker for breast cancer and gastric cancer progression in microarray and proteomics studies [42,43]. Previous investigations of canonical H2A isoforms found that up-regulation of specific H2A sequences in chronic lymphocytic leukemia and in MCF-7 breast cancer cells caused increased proliferation and carcinogenesis in those cell types [44,45]. Similar studies should be performed by overexpressing H2B1B and H2B1M in some of the cancer cells where the levels of these variants are very low to see if this causes a decrease in cancer cell proliferation or progression.

We demonstrated that polyadenylated H2B mRNA is produced independent of DNA replication. In this manner, the H2B1D, H2B1K, and H2B2E isoforms that produce polyadenylated transcripts are similar to replication-independent variants. However, histone production is not independent of DNA replication at the protein level. After blocking DNA synthesis in S-phase using a double-thymidine block, there was very little synthesis of the H2B1D protein despite continued production of its transcript. It is possible that the polyadenylated transcript level is very low and does not contribute significantly to the total H2B protein level. In fact, a previous study on polyadenylated H2B2E found that polyadenylated H2B2E transcript levels were very low in normal cycling cells [38]. If this is true, and the amount of protein being produced outside of DNA replication is very low, then we may not be able to measure the increase in H2B from replication-independent production. It is also possible that the polyadenylated H2B transcripts are not translated, and are instead the result of aberrant histone 3′ end processing as a result of missing cell-cycle-dependent histone mRNA processing proteins [39,46,47], and that most histone production in non-cycling cells comes from some basal histone transcription that occurs throughout the cell cycle. In non-cycling myotubes, all histone H2B isoforms turned over at the same rate, suggesting no preference for any sequence, regardless of whether it can be polyadenylated or not. On the other hand, histone variants with genes outside of the main clusters (for example H2A.Z and H1.x) had different turnover profiles compared to the replication-dependent variants in myoblasts and myotubes, suggesting that there may be specific regulation for these variants in myotubes.

Out of the three polyadenylated H2B isoforms, we were only able to distinguish H2B1D from other H2B isoforms using its N-terminal tail sequence and thus it was the only form that could be quantified independently using bottom-up MS. H2B1K and H2B2E have the same N-terminal tail sequence as H2B1C, and these variants can only be distinguished from other H2B by top-down mass spectrometry using instruments with a higher mass resolution than what is available in our lab, but it would be interesting to see if they show the same trends as H2B1D. In any case, H2B produced outside of the cell cycle in HeLa cells is not as abundant as known H2A variants and probably does not have the same set of important replication-independent functions.

## Conclusions

We identified all 13 somatic H2B isoforms using a combination of different mass spectrometry methods in the most comprehensive study of H2B isoforms and their modifications to date. In addition, we developed a method to quantify nine of the H2B isoforms using middle-down MS. We applied this method to determine whether any H2B isoforms have variations in expression levels across cell types, are produced independently of cell cycle, or are associated with specific interacting partners. We found that the level of histone H2B isoforms, particularly H2B1B and H2B1M, varies across cancer cell lines, suggesting that H2B expression may be regulated differently across cell types or possibly misregulated in cancer. We also found that some H2B isoform genes can produce polyadenylated mRNA independent of the cell cycle, but these transcripts do to seem to be translated in significant quantities outside of S-phase.

## Methods

### Cell culture

HeLa S3 cells were maintained in suspension in minimal essential Joklik’s modification medium (Sigma Aldrich, St. Louis, MO, USA) with 10% newborn calf serum (Invitrogen, Carlsbad, CA, USA) and penicillin-streptomycin (pen-strep) solution diluted 1:100 (10,000 units penicillin G and 10 mg streptomycin per milliliter) (Thermo Fisher Scientific, Waltham, MA, USA) and supplemented with 1% Glutamax (Invitrogen, Carlsbad, CA, USA). Jurkat, and HL60, cells were maintained in RPMI-1640 (Mediatech, Inc., Manassas, VA, USA) medium supplemented with 10% fetal bovine serum and 1:100 pen-strep, and PANC cells and U2OS cells were grown in Dulbecco’s Modified Eagle’s Medium (DMEM; Invitrogen, Carlsbad, CA, USA) supplemented with 10% fetal bovine serum and 1:100 pen-strep, and PC3 cells were grown in Ham’s F12-K media (Invitrogen, Carlsbad, CA, USA) supplemented with 10% fetal bovine serum and 1:100 pen-strep. Data for the remaining cell lines were analyzed using RAW data from a previous study in our lab. Cell growth conditions, sample preparation, and mass spectrometer parameters for the remaining cell lines can be found in Leroy *et al*. (141).

For the cell cycle synchronization experiments, HeLa cells were blocked in S-phase by adding 2 mM thymidine to cells growing at a density of 3 × 10^5^ cells/mL. The thymidine was removed for 9 h, followed by a second 17-h block in 2 mM thymidine. Following synchronization, cells were collected at 2, 4, 6, and 8 h and fixed in ethanol and stained with propidium iodide for cell cycle analysis by flow cytometry or snap-frozen and saved for q-RT-PCR analysis.

For the cell cycle SILAC-labeling experiments, HeLa cells were maintained in heavy arginine media with 10% dialyzed fetal bovine serum (FBS) and penicillin-streptomycin solution diluted 1:100. The media was made in-house using Joklik’s modification of Minimal Essential Media Eagle formulation, replacing arginine in the media with the same concentration of stable-isotope-labeled arginine (L-Arginine:HCl, ^13^C_6_,^15^ N_4_, Cambridge Isotope Laboratories, Andover, MA, USA). The cells were maintained in heavy media for 24 h and then blocked in S-phase by adding 2 mM thymidine to the growth medium. Cells were collected at time points before the thymidine block and up to 24 h after the block to monitor heavy arginine incorporation into histones.

Human myogenic cell line, LHCN M2, was a kind gift from Dr. Woodring Wright (UT Southwestern Medical Center at Dallas, Dallas, TX, USA). The immortalized myoblasts were cultured in proliferation medium and, once confluent, were differentiated in low-serum-containing medium as described previously [48]. For heavy arginine labeling of myoblasts and myotubes, the media-contained DMEM was exchanged for one deficient in arginine and lysine (Thermo Scientific, Waltham, MA, USA) supplemented with [13C6] L-Arginine (98% purity) (Cambridge Isotope Laboratories, Tewksbury, MA, USA) and light lysine and proline (Sigma Aldrich, St. Louis, MO, USA). Similarly, dialyzed FBS (Invitrogen, Carlsbad, CA, USA) was used instead of FBS (Invitrogen, Carlsbad, CA, USA). Briefly, myoblasts grown in light proliferation medium (day 0) were divided into two batches, one expanded in heavy medium and the other in light medium. Cells grown in heavy proliferation medium were harvested on days 1, 2, 3, and 7. Cells that grew confluent in light proliferation medium (7 days) were switched to heavy differentiation medium and harvested on days 1, 2, 3, and 7 using trypsin after washing with sterile PBS. The cells were collected by centrifugation, washed in PBS, snap-frozen in liquid nitrogen, and stored at −80°C. Two biological replicates were used for histone analysis.

LHCN M2 cells were fixed 4 days after seeding for myoblasts and 6 days after differentiation for myotubes, and indirect immunofluorescence staining was performed using 1:20 dilution of myosin heavy chain supernatant (Developmental Studies Hybridoma Bank, cat# MF-20) as primary antibody and anti-mouse Alexa Fluor 488 as secondary antibody. The images were visualized at 20× in Nikon Inverted Microscope TE2000 and acquired with Image Pro Plus 7.0, (Media Cybernetics, Rockville, MD, USA).

### q-RT-PCR

RNA was extracted from synchronized cells using TRIzol (Invitrogen, Carlsbad, CA, USA). Five micrograms of RNA was then treated with DNase I (NEB, Ipswich, MA, USA) at 65°C for 1 h to remove contaminating genomic DNA. cDNA was synthesized using random hexamer primers and the Superscript III kit (Invitrogen, Carlsbad, CA, USA). H2B isoforms were then quantified in each sample using isoform-specific primers and Sybrgreen master mix (Invitrogen, Carlsbad, CA, USA). Two housekeeping genes (18S RNA and glyceraldehyde 3-phosphate dehydrogenase (GAPDH)) were included in cell cycle analyses, and three housekeeping genes (18S RNA, RORα, and GAPDH) were included for normalization (primer sequences available upon request). PCR was performed using 100 nM primers and the following cycling conditions: 50°C for 2 min, 95°C for 10 min, followed by 40 cycles of 95°C for 15 s and 59°C for 1 min. A no-RT control was also used to ensure that there was no contaminating DNA in the RT reaction; this is important because H2B genes do not contain introns and so have the same sequence as cDNA.

### Histone extraction

Cells were harvested and washed twice using phosphate-buffered saline. The cells were incubated in 10× the cell pellet volume of nuclear isolation buffer (15 mM Tris–HCl, 15 mM NaCl, 60 mM KCl, 5 mM MgCl_2_, 1 mM CaCl_2_, 250 mM sucrose, pH 7.5, and 0.5 mM AEBSF, 10 mM sodium butyrate, 2.5 μM microcystein, 1 mM DTT added fresh) with 0.3% NP-40 on ice for 5 min. The nuclei were collected by centrifuging in a table-top centrifuge at 500 × *g* at 4°C for 5 min. The resulting nuclear pellet was washed twice with nuclear isolation buffer without NP-40. After extracting the nuclei, histones were extracted by re-suspending the nuclei in 5× the nuclear pellet volume of 0.4 N sulfuric acid at 4°C with rotation. The insoluble nuclear debris were pelleted at 3600 × *g* at 4°C for 5 min, and the supernatant was retained. Finally, histone proteins were precipitated on ice for 1 h after adding 100% trichloroacetic acid to the acid extraction supernatant at a ratio of 1:5 (*v*/*v*). The precipitated proteins were washed once with 0.1% HCl in acetone (−20°C), and then twice with acetone (−20°C), then dried overnight on the bench-top.

### RP-HPLC purification of histone H2B

Around 500 μg of acid-extracted histones were re-suspended in 200 μL RP-HPLC buffer A (5% HPLC-grade acetonitrile, 95% HPLC-grade water, 0.2% trifluoroacetic acid (TFA)) then loaded onto a Vydac 4.6 mm, 5 μm, 300 Å pore size column for 5 min at 2% RP-HPLC buffer B (95% HPLC-grade acetonitrile, 5% HPLC-grade water, 0.2% TFA) and resolved using a gradient from 30% buffer B to 60% buffer B at a flow rate of 0.8 μL/min over 100 min. Fractions corresponding to histone H2B were pooled and immediately dried in a speed-vac.

### Propionic anhydride derivatization

Acid-extracted histones or HPLC-purified H2B were re-suspended in 20 μL 100 mM ammonium bicarbonate (pH 8), and the final pH was adjusted to 8 using ammonium hydroxide (28% to 30% ammonia basis, Sigma Aldrich, St. Louis, MO, USA). The starting pH for the propionic anhydride derivatization should be pH 8 and not higher (cause of unwanted serine propionylation) or lower (decreased reaction efficiency) to ensure that only primary amines (lysines and protein N-termini) are derivatized. After samples were set to pH 8, they were mixed with 10 μL of a freshly prepared propionic anhydride solution (75% isopropanol, 25% propionic anhydride) by vortexing, and then, 5 μL of ammonium hydroxide was immediately added to the mixture. More ammonium hydroxide was added if the pH needed to be adjusted to pH 8, then the samples were incubated at 37°C for 10 min. After incubation, the samples were dried in a speed-vac to <10 μL to remove propionic acid formed as a result of the reaction. The propionic anhydride derivatization reaction was repeated one more time. After the samples were dried to <10 μL, they were brought up to 50 μL using 10 mM ammonium bicarbonate (pH 8) and digested with trypsin at a ratio of 1 μg trypsin:20 μg sample for 6 to 8 h at 37°C. After the trypsin digest, the samples were dried to 20 μL and subjected to two more rounds of propionylation to cap peptide N-terminal amines. Following propionylation, the samples were de-salted using C18-StageTips [49] in preparation for MS analysis.

### Top-down H2B MS analysis

A linear triple quadrupole orbitrap (LTQ-Orbitrap) XL mass analyzer (Thermo Fisher Scientific, Waltham, MA, USA) was used for the initial top-down MS analyses of HeLa and Jurkat H2B. The LTQ-Orbitrap XL was set to have an automatic gain control (AGC) value of 5 × 10^5^ for the FT full-MS scans and the FT MS/MS scans, the HCD gas and the ‘FT zero offset’ were turned off, and the detection delay was set to ‘low.’ Next, the LTQ ion optics were tuned on the myoglobin 848.64 m/z ion to improve ion transmission for large ions. The resolving power of the Orbitrap was set to 30,000.

Top-down H2B analysis of other cancer cell lines was performed on an Orbitrap Fusion instrument (Thermo Fisher Scientific, Waltham, MA, USA). The AGC target was set to 5 × 10^5^, and the maximum injection time was 100 ms, for the Orbitrap when it was collecting both full-MS and MS/MS spectra. A resolving power of 60,000 was used for full-MS scans and 30,000 for MS/MS scans. The S lens voltage was set at 60%, and mild in-source fragmentation (around 40 V) was used to improve signal for the high-m/z range and to reduce non-covalent adducts.

Pure histone H2B at a concentration of 1 μg/μL was infused onto the mass spectrometer using a pulled, fused silica tip (360-μM outer diameter, 50-μm inner diameter) on a nano-ESI source at a flow rate of 0.7 μL/min using a 50-μL syringe. The source voltage was set to 2.3 V for both instruments. The histone H2B signal was averaged over 10 microscans, and the scan range was set to encompass the most abundant charge states of histone H2B. After recording the full-MS spectra for several minutes, H2B peaks were selected for fragmentation using an isolation window of 3 m/z with no fragmentation to record which species were being successfully isolated. Next, the ETD reaction time was increased from 5 ms until the optimal MS/MS signal and a wide range of fragment ion masses were achieved. ETD spectra were summed over 5 microscans, and the resulting spectra were averaged over several minutes of collection time to improve the signal.

### Bottom-up LC-MS analysis

Samples were re-suspended in buffer A (0.1% formic acid in LC/MS-grade water). Chromatographic separation of propionylated, digested histone or pure H2B samples was performed on a 15-cm reversed-phase column packed with Magic C18 AQ resin (5 μm, 100 Å pore size, Michrom Bioresources, Auburn, CA, USA) using a flow rate of 0.25 μL/min on either an Easy-nanoLC (Thermo Fisher Scientific, Waltham, MA, USA) or an Eksigent nanoLC-Ultra 2D using a gradient from 1% buffer B (0.1% formic acid in LC/MS-grade acetonitrile) to 32% B over 42 min. The HPLC was coupled to either an LTQ-Orbitrap Velos or an LTQ-Orbitrap Elite. The mass spectrometer was operated in a semi-targeted mode over three segments of the run. During the first two segments, one full-MS spectrum was collected from 350 to 1,600 m/z, then five masses corresponding to histone H4 and H3 acetylation states were targeted, and the remaining scans were data-dependent CAD MS/MS scans of the top six most abundant masses from the full-MS scan. In the third segment, two full-MS scans were collected: one from 350 to 1,600 m/z and one from 900 to 1,300 m/z, followed by data-dependent scans of the top ten most abundant ions from the second, limited mass range full-MS scan. The AGC settings for the mass spectrometers were set as follows: 3 × 10^5^ for the ion trap and 1 × 10^6^ for the Orbitrap. The relative abundance of histone H2B isoforms and their acetylated forms was calculated based on the area under the extracted ion chromatograms from the limited mass-range MS1 scan for all of the detectable charge states of each H2B peptide.

RAW files of heavy-arginine-labeled histone samples were loaded into Proteome Discoverer (version 1.4) for analysis. The data was searched against a custom database of 82 known human histone H3, H4, H2A, H2B, and H1 and other chromatin-related protein sequences using Mascot (version 2.2, Matrix Science, Boston, MA, USA) in Proteome Discoverer. We set N-terminal propionylation as a static modification and lysine propionylation and acetylation as variable modifications. The enzyme was set as Arg-C, and up to two missed cleavages were allowed. Quantification was done through the *event detector* and *precursor ion quantifier* nodes of Proteome Discoverer. The search results and peak matches were validated manually for H2B spectra using MS/MS spectra and relative retention times as a guide, and some false-positive peptide identifications were excluded from the analysis. Results from each peptide were averaged. Only peptides that are unique to a particular histone sequence and that are known to not be heavily modified were used in our analyses.

## Additional files

Additional file 1: Table S1. Jurkat H2B isoform masses and abundances detected by top-down MS. All of the H2B isoforms expressed in human somatic cells are the same length and have very few points of sequence variation. Consequently, many of them have the same intact mass or masses that are similar enough that they cannot be resolved based on the full-MS spectra alone. Additional file 2: Figure S1. Characteristic proline-directed y ion fragments in H2B(1–29) CAD spectra can be used to distinguish sequences and PTM states. Characteristic proline-directed CAD fragment ions (y20, y22, y23, y24) can be used to distinguish between isobaric peptides (A) H2B1D(1 ac) and (B) H2B1N. ac = acetylation. Additional file 3: Figure S2. Levels of acetylation on H2B isoform N-terminal tails. The abundance of each modification for a peptide was normalized against the total abundance of that peptide. Mean ± SE, *n* = 5. Additional file 4: Figure S3. Alignment of the intact mass spectra of RP-HPLC-purified H2B from different cancer cell lines. Top-down MS was used to confirm that there are differences in the levels of H2B isoforms across cancer cell lines. Additional file 5: Figure S4. Confirmation of cells synchronization by FACS analysis. Cells in G1-phase of the cell cycle have half the DNA and therefore half of the propidium iodide staining of cells in G2/M-phase. Over 90% of cells were synchronized at each cell cycle phase. Additional file 6: Figure S5. Immunofluorescence microscopy images (20×) of human myoblast cell line, LHCN M2, in proliferation medium (A) and after culture in differentiation medium for 4 days (B). Actively cycling myoblasts do not express myosin heavy chain (MHC) while on differentiation, cells exit cell cycle, fuse into myotubes containing multiple nuclei, and express MHC (Alexa Fluor-488, green). Additional file 7: Figure S6. Comparison of H2B variant levels in myoblasts versus myotubes by qPCR. (A) Relative fold difference of histone mRNA in myoblasts (Prolif) versus myotubes (Diff). (B) Polyadenylated H2B1D (H2B1DPa) mRNA fold difference in myoblasts and myotubes. This is the same data as in (A), except with a restricted *y*-axis to better visualize the results. The DCt values were calculated by normalizing histone Ct values to the geometric mean of three housekeeping genes (18S RNA, GAPDH, RORα). Replicate 1 and Replicate 2 are biological replicates. Mean ± SD, *n* = 3. Additional file 8: Figure S7. Histone H2A and H1 variant levels in myoblast cells compared to myotube cells. Proliferating myoblast (prolif) and differentiated myotube cells (diff) were cultured in media containing heavy arginine, and samples were collected over the course of 7 days to monitor new histone synthesis. Heavy (new) over light (old) levels for histone variants in proliferating or differentiated cells were plotted over time for two biological replicates.

## Abbreviations

Ac: acetylation
AGC: automatic gain control
CAD: collisionally activated dissociation
ETD: electron transfer dissociation
G: glycosylation
HCD: higher energy collisional dissociation
hESC: human embryonic stem cell
HFF: human foreskin fibroblast
LTQ-Orbitrap: linear triple quadrupole orbitrap
Me: methylation
SILAC: stable isotope labeling of amino acids in cell culture
TFA: trifluoroacetic acid
Ub: ubiquitination
