# Supplementary material for: Multi-faceted quantitative proteomics analysis of histone H2B isoforms and their modifications
Source: Epigenetics Chromatin. 2015 Apr 22;8:15. doi: 10.1186/s13072-015-0006-8 (PMC4411797; doi:10.1186/s13072-015-0006-8)
Supplement: Additional file 1: Table S1. — Jurkat H2B isoform masses and abundances detected by top-down MS. All of the H2B isoforms expressed in human somatic cells are the same length and have very few points of sequence variation. Consequently, many of them have the same intact mass or masses that are similar enough that they cannot be resolved based on the full-MS spectra alone. [file 13072_2015_6_MOESM1_ESM.docx]

**Supplementary Table 1:** Jurkat H2B isoform masses and abundances detected by Top-down MS

| **Isoform** | **Theoretical mass (Da)** | **Observed mass (Da)** | **Δ Mass (Da)** | **PIRR** | **m/z** | **Charge** | **Score** |
| --- | --- | --- | --- | --- | --- | --- | --- |
| H2B1H | 13752.501 | 13750.525 | -1.98 | 0.11 | 765.370 | 18 | 96% |
| H2B1K | 13750.522 |  | 0.003 |  |  |  |  |
| H2B1C | 13766.517 | 13765.541 | -0.98 | 0.27 | 766.205 | 18 | 98% |
| H2B1O | 13766.517 |  | -0.98 |  |  |  |  |
| H2B1J | 13764.538 |  | 1.00 |  |  |  |  |
| H2B2F | 13780.533 | 13780.556 | 0.02 | 0.20 | 767.039 | 18 | 95% |
| H2B2E | 13780.533 |  | 0.02 |  |  |  |  |
| H2B1D | 13796.528 | 13796.546 | 0.02 | 0.13 | 767.927 | 18 | 92% |
| H2B1C 1ac | 13808.528 | 13807.563 | -0.96 | 0.07 | 768.539 | 18 | 73% |
| H2B1B | 13810.543 |  | -2.98 |  |  |  |  |
| H2B1M | 13849.591 | 13848.515 | -1.08 | 0.10 | 770.814 | 18 | 89% |
| H2B1C 2ac | 13850.537 |  | -2.02 |  |  |  |  |
| H2B2F 1ac | 13822.544 | 13816.510 | -6.03 | 0.09 | 769.036 | 18 | 79% |
| H2B1D 1ac | 13838.539 | 13836.521 | -2.02 | 0.04 | 770.148 | 18 | 76% |
| H2B1L | 13812.559 | ND |  |  |  |  |  |
| H2B3B | 13768.496 | ND |  |  |  |  |  |
