# Supplementary figures and images for: Multi-faceted quantitative proteomics analysis of histone H2B isoforms and their modifications
Source: Epigenetics Chromatin. 2015 Apr 22;8:15. doi: 10.1186/s13072-015-0006-8 (PMC4411797; doi:10.1186/s13072-015-0006-8)

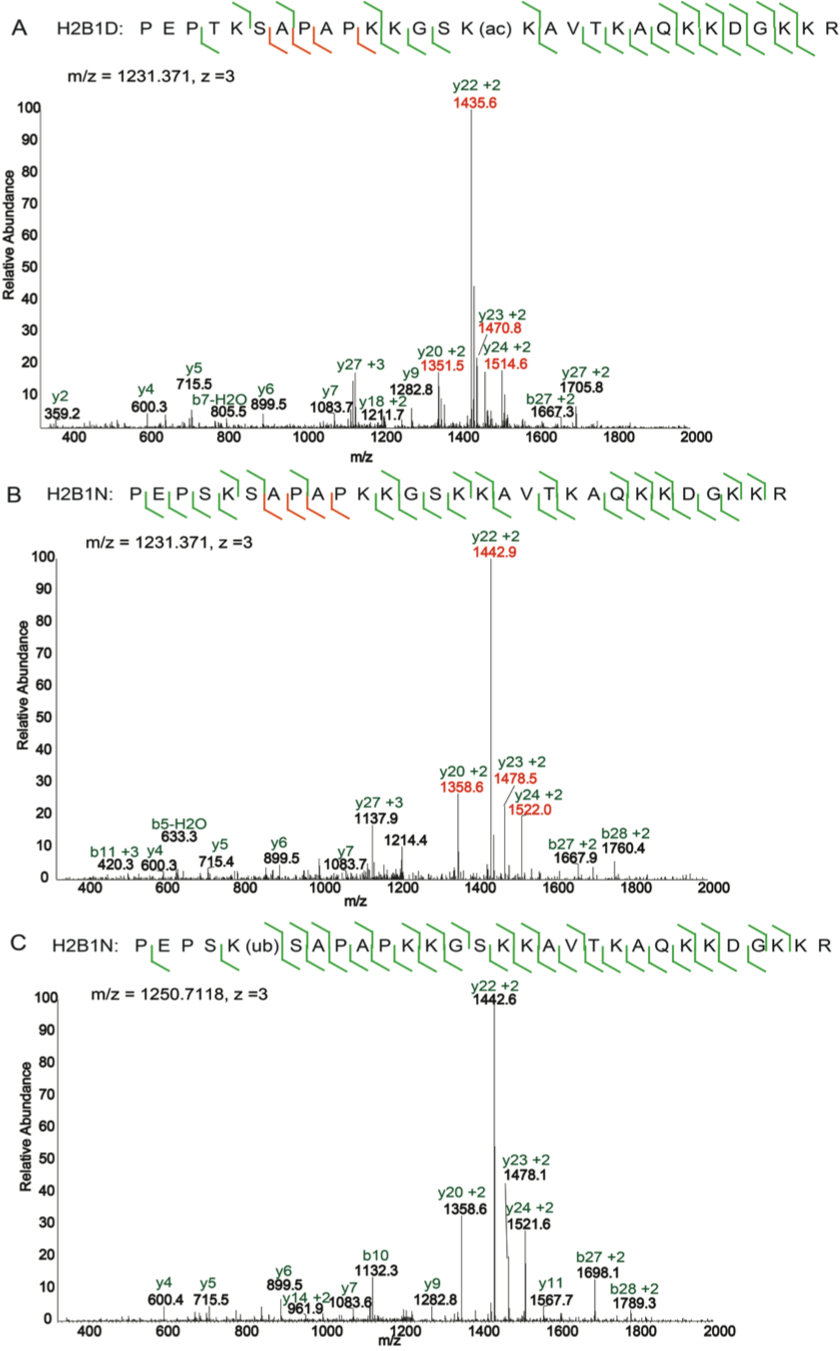

Supplement: Additional file 2: Figure S1. — Characteristic proline-directed y ion fragments in H2B(1–29) CAD spectra can be used to distinguish sequences and PTM states. Characteristic proline-directed CAD fragment ions (y20, y22, y23, y24) can be used to distinguish between isobaric peptides (A) H2B1D(1 ac) and (B) H2B1N. ac = acetylation. [file 13072_2015_6_MOESM2_ESM.png]

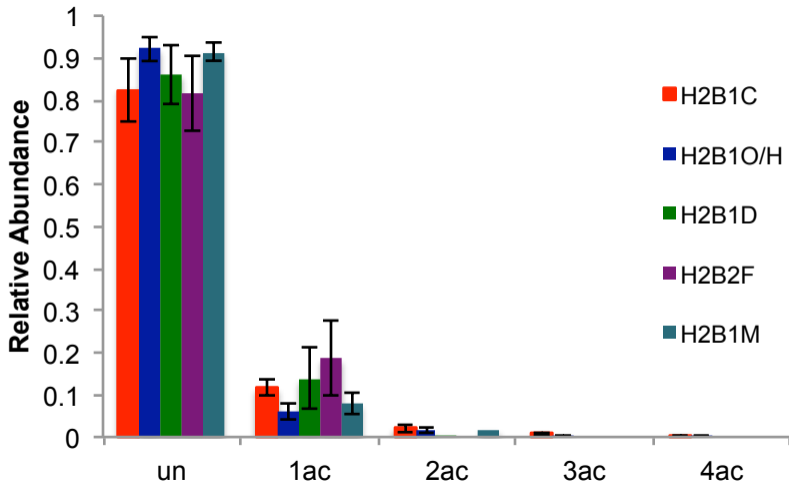

Supplement: Additional file 3: Figure S2. — Levels of acetylation on H2B isoform N-terminal tails. The abundance of each modification for a peptide was normalized against the total abundance of that peptide. Mean ± SE, n = 5. [file 13072_2015_6_MOESM3_ESM.pdf]

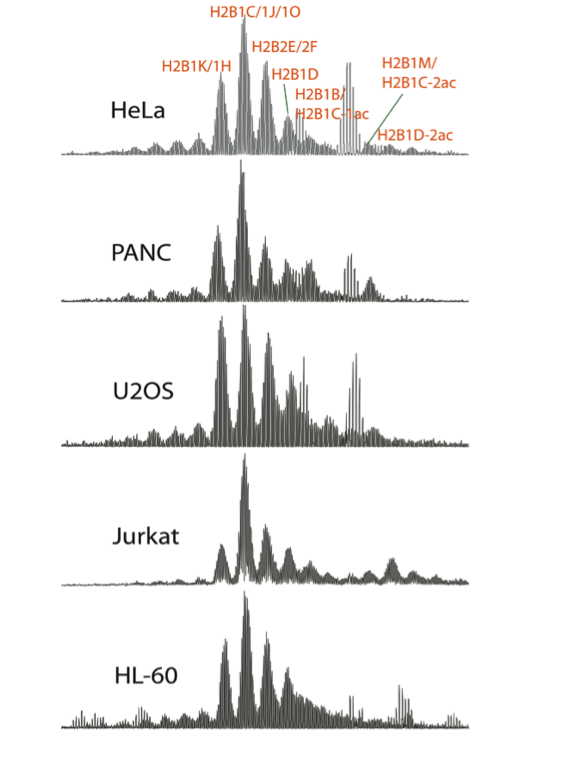

Supplement: Additional file 4: Figure S3. — Alignment of the intact mass spectra of RP-HPLC-purified H2B from different cancer cell lines. Top-down MS was used to confirm that there are differences in the levels of H2B isoforms across cancer cell lines. [file 13072_2015_6_MOESM4_ESM.png]

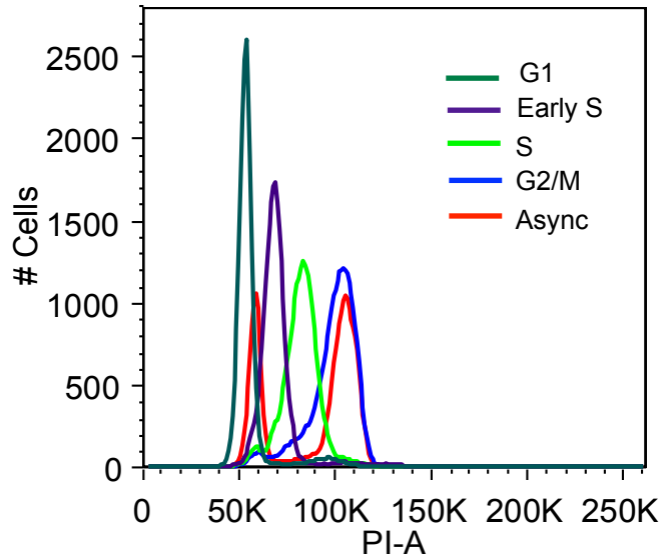

Supplement: Additional file 5: Figure S4. — Confirmation of cells synchronization by FACS analysis. Cells in G1-phase of the cell cycle have half the DNA and therefore half of the propidium iodide staining of cells in G2/M-phase. Over 90% of cells were synchronized at each cell cycle phase. [file 13072_2015_6_MOESM5_ESM.pdf]

**A**

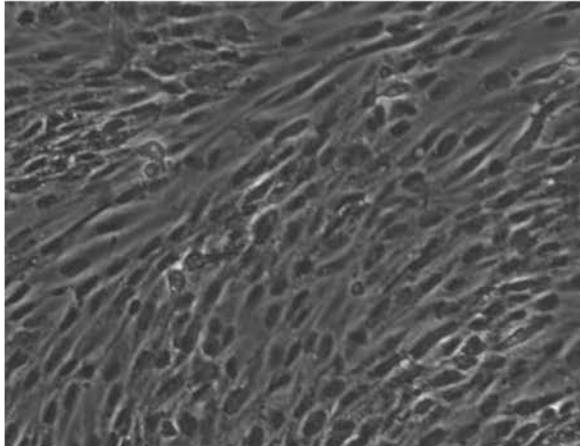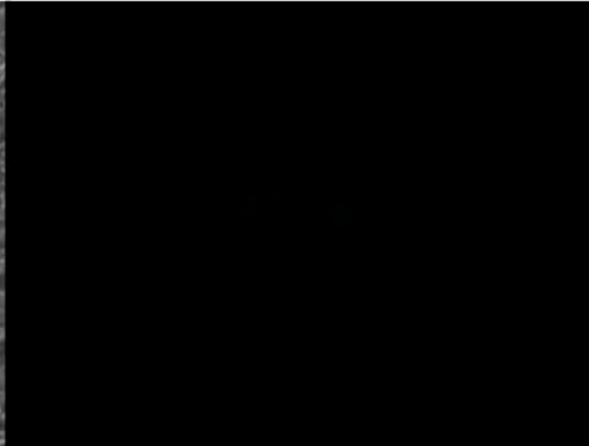

**B**

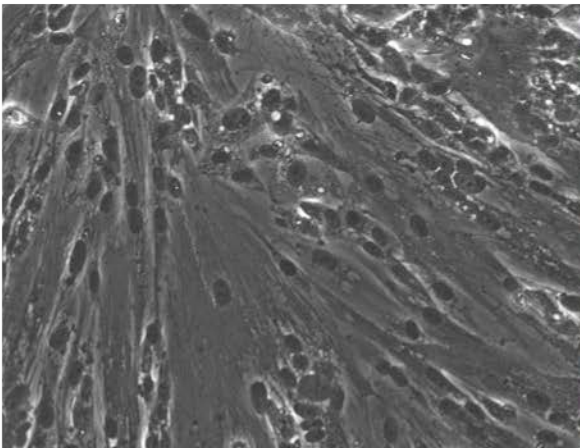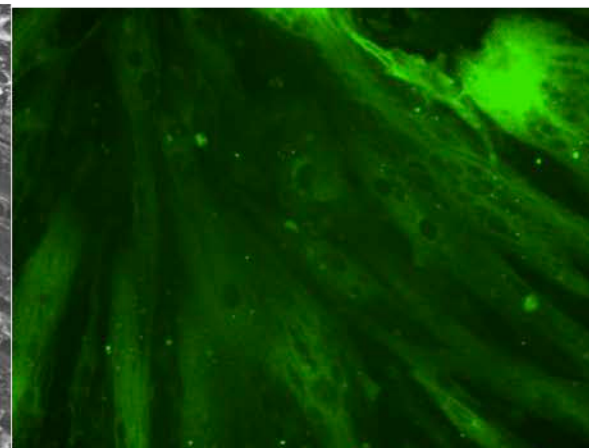

Proliferating  
myoblasts

Differentiated  
myotubes

Supplement: Additional file 6: Figure S5. — Immunofluorescence microscopy images (20×) of human myoblast cell line, LHCN M2, in proliferation medium (A) and after culture in differentiation medium for 4 days (B). Actively cycling myoblasts do not express myosin heavy chain (MHC) while on differentiation, cells exit cell cycle, fuse into myotubes containing multiple nuclei, and express MHC (Alexa Fluor-488, green). [file 13072_2015_6_MOESM6_ESM.pdf]

A

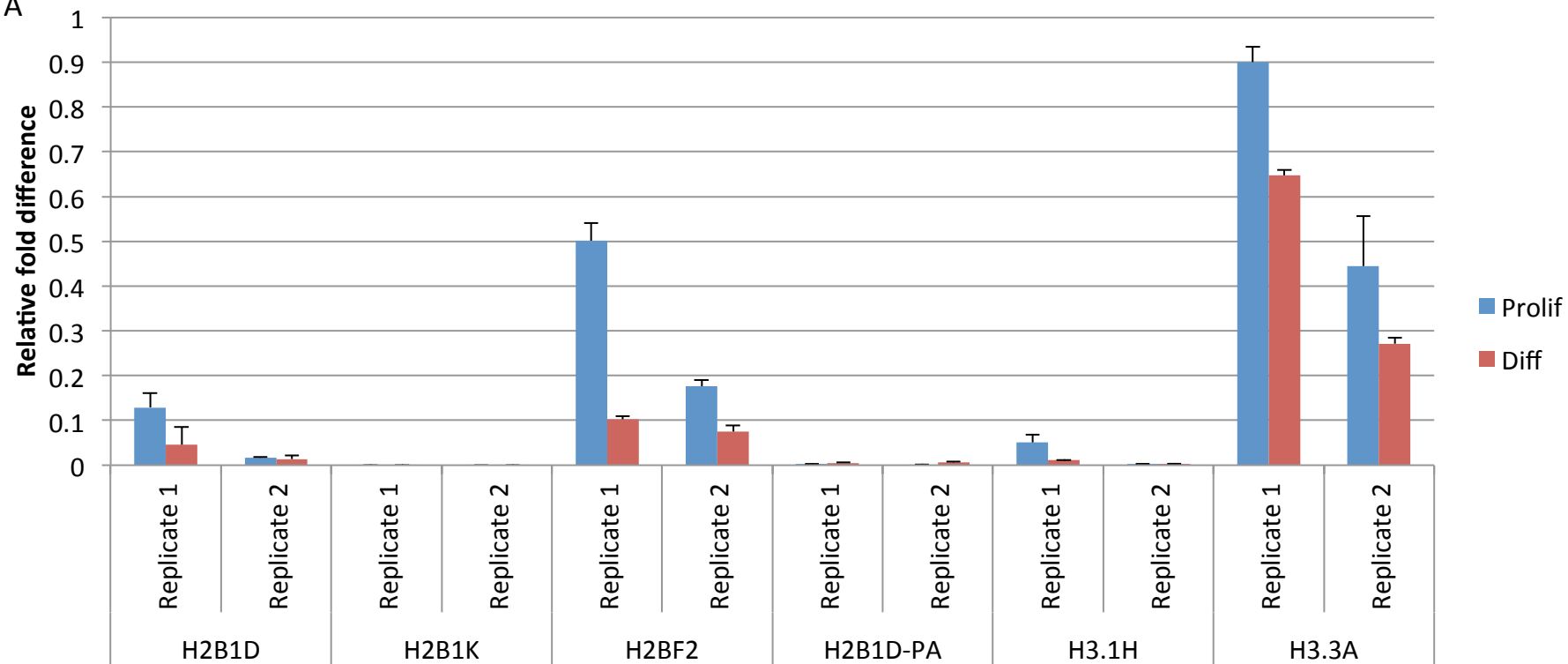

B

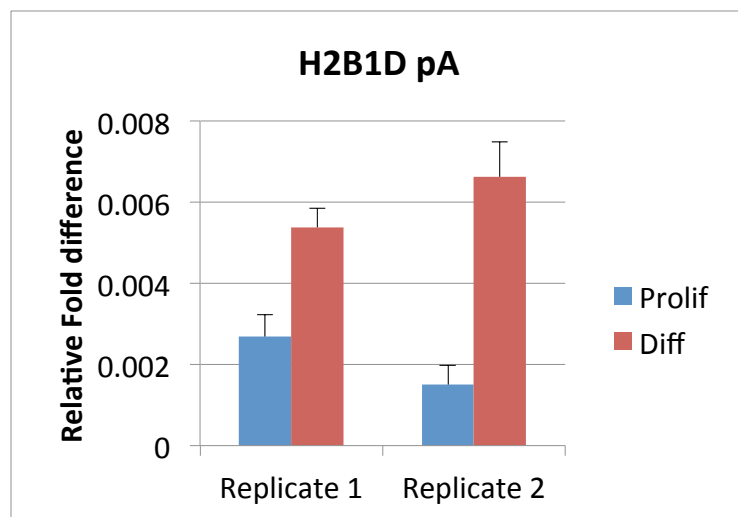

Supplement: Additional file 7: Figure S6. — Comparison of H2B variant levels in myoblasts versus myotubes by qPCR. (A) Relative fold difference of histone mRNA in myoblasts (Prolif) versus myotubes (Diff). (B) Polyadenylated H2B1D (H2B1DPa) mRNA fold difference in myoblasts and myotubes. This is the same data as in (A), except with a restricted y-axis to better visualize the results. The DCt values were calculated by normalizing histone Ct values to the geometric mean of three housekeeping genes (18S RNA, GAPDH, RORα). Replicate 1 and Replicate 2 are biological replicates. Mean ± SD, n = 3. [file 13072_2015_6_MOESM7_ESM.pdf]

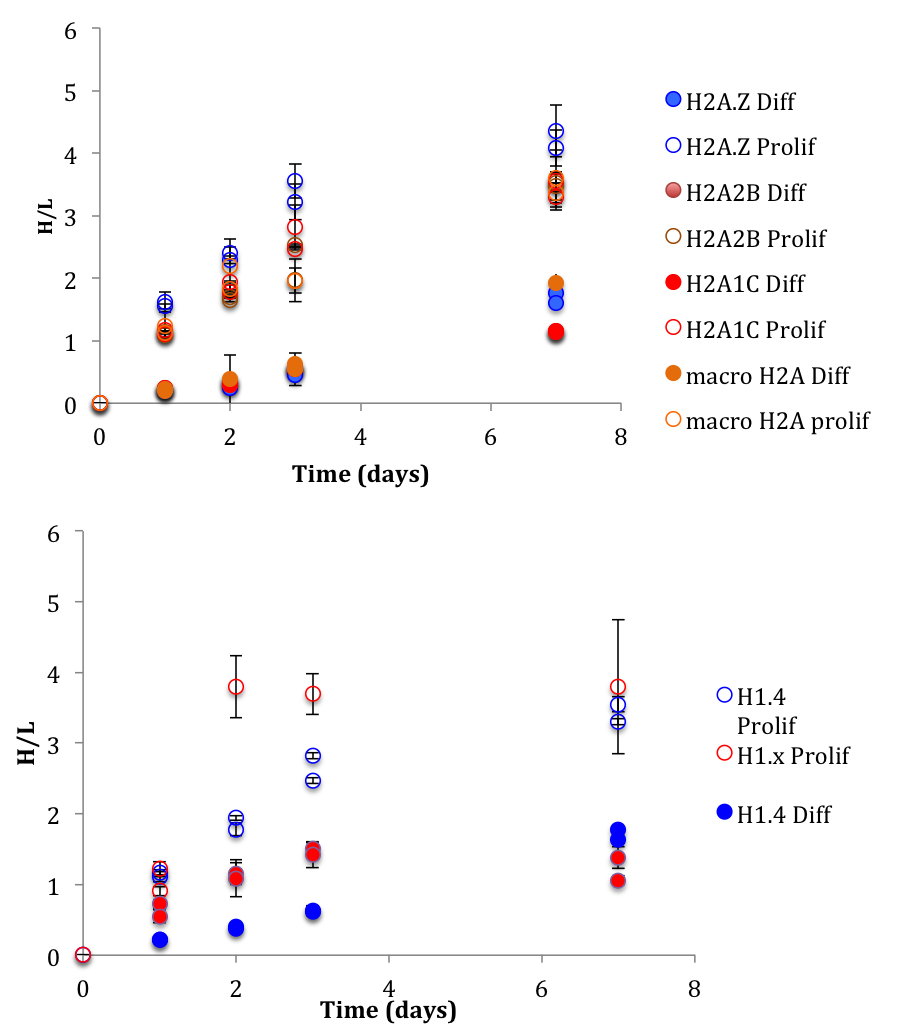

Supplement: Additional file 8: Figure S7. — Histone H2A and H1 variant levels in myoblast cells compared to myotube cells. Proliferating myoblast (prolif) and differentiated myotube cells (diff) were cultured in media containing heavy arginine, and samples were collected over the course of 7 days to monitor new histone synthesis. Heavy (new) over light (old) levels for histone variants in proliferating or differentiated cells were plotted over time for two biological replicates. [file 13072_2015_6_MOESM8_ESM.png]
